# The Efficacy and Mechanism of Chinese Herbal Medicine on Diabetic Kidney Disease

**DOI:** 10.1155/2019/2697672

**Published:** 2019-08-25

**Authors:** Zhenzhen Lu, Yifei Zhong, Wangyi Liu, Ling Xiang, Yueyi Deng

**Affiliations:** The Department of Nephrology, Longhua Hospital, Shanghai University of Traditional Chinese Medicine, Shanghai 200032, China

## Abstract

Diabetic kidney disease (DKD) is the most common microvascular complication of diabetes and is one of the main causes of end-stage renal disease (ESRD) in many countries. The pathological features of DKD are the hypertrophy of mesangial cells, apoptosis of podocytes, glomerular basement membrane (GBM) thickening, accumulation of extracellular matrix (ECM), glomerular sclerosis, and tubulointerstitial fibrosis. The etiology of DKD is very complicated and many factors are involved, such as genetic factors, hyperglycemia, hypertension, hyperlipidemia, abnormalities of renal hemodynamics, and metabolism of vasoactive substances. Although some achievements have been made in the exploration of the pathogenesis of DKD, the currently available clinical treatment methods are still not completely effective in preventing the progress of DKD to ESRD. CHM composed of natural products has traditionally been used for symptom relief, which may offer new insights into therapeutic development of DKD. We will summarize the progress of Chinese herbal medicine (CHM) in the treatment of DKD from two aspects. In clinical trials, the Chinese herbal formulas were efficacy and safety confirmed by the randomized controlled trials. In terms of experimental research, studies provided evidence for the efficacy of CHM from the perspectives of balancing metabolic disorders, reducing inflammatory response and oxidative stress, antifibrosis, protecting renal innate cells, and regulating microRNA and metabolism. CHM consisting of different ingredients may play a role in synergistic interactions and multiple target points in the treatment of DKD.

## 1. Introduction

**Figure 1:**
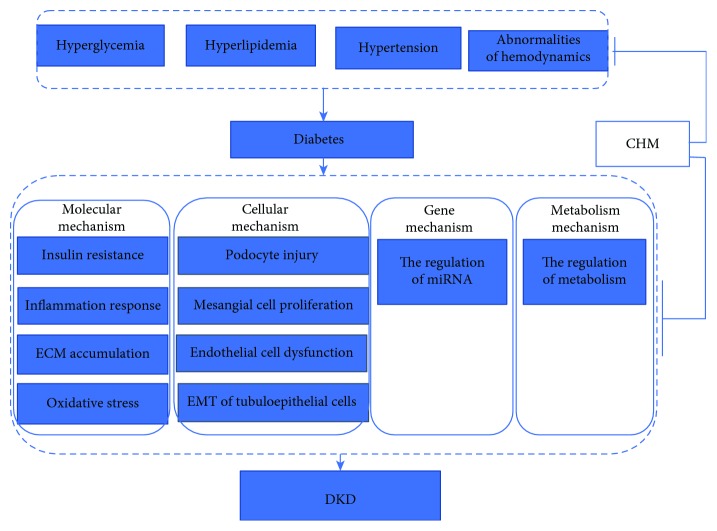
Traditional Chinese medicine treatment of diabetic nephropathy from molecular, cellular, and gene levels. Abbreviations: ECM: extracellular matrix; EMT: epithelial-mesenchymal transition.

Diabetic kidney disease (DKD) refers to kidney damage caused by diabetes, based on the appearance of proteinuria in diabetic patients. Clinically, the differential diagnosis of diabetic nephropathy and nondiabetic nephropathy mainly depends on the history of diabetes, screening of urinary protein, retinopathy and neuropathy, and so on. When it is difficult to diagnose, it depends on kidney biopsies. Epidemiological studies have shown that global burden of diabetes now affects more than 425 million people. If nothing is done, the number of people with diabetes worldwide will rise to 629 million in 2045 [1]. With the prevalence in diabetes, the incidence of DKD is growing rapidly. About 30%-40% of diabetic patients develop DKD, and one third of the patients further progress to end-stage renal disease (ESRD), which brings enormous economic burden for our society [2, 3]. DKD is divided into five stages according to Mogensen criteria by the course of DKD, pathological changes, the degree of proteinuria, and renal dysfunction. Persistent albuminuria and renal injury are well-established clinical signature and risk factors of renal lesions, which cause glomerulosclerosis and subsequent interstitial fibrosis [4]. Early medical research has shown that angiotensin-converting enzyme inhibitors (ACEIs) and angiotensin II receptor blockers (ARBs), the first-line treatment for DKD, can reduce proteinuria and have a certain effect on delaying the progress of renal dysfunction [5]. However, the side effects of ACEI/ARB, such as dry cough, hypotension, hyperkalemia, and angioedema, limit the application of these drugs. Evidence-based medical studies have shown that these agents have not significantly reduced DKD vascular event rate and mortality [6]. Some promising therapies addressing novel targets, such as incretin-based therapies glucagon-like peptide-1 (GLP-1) receptor antagonists and dipeptidyl peptidase-4 (DPP-4) inhibitors, might improve albuminuria in type 2 diabetes, while effects on clinically relevant kidney outcomeshile effects on clinically relevant kidney outcomes are still under evaluation [7]. Several large-scale trials focused on sodium-glucose cotransporter-2 (SGLT2) inhibitors (SGLT2i) have reported favorable effects on the primary endpoint, a composite of myocardial infarction, stroke, and cardiovascular death of people who have type 2 diabetes [8, 9]. Recently, CREDENCE clinical trials, a double-blind, randomized trial, demonstrated that patents in the canagliflozin group have a lower risk of the primary composite outcome of end-stage kidney disease, doubling of the serum creatinine level or death from renal or cardiovascular causes than in the placebo group with a median follow-up of 2.62 years [10]. However, adverse events associated with SGLT2 inhibitors include genital mycotic infections, urinary tract infection, hypoglycemia, diabetic ketoacidosis, hypotension, acute kidney injury, fractures, and amputations [11]. It is of great urgency to search for safe and effective therapies for DKD. A systematic review and meta-analysis reported that traditional Chinese medicine (TCM) had a great beneficial effect on the reduction of urinary albumin creatinine ratio and proteinuria [12]. TCM has formed a unique system to diagnose and cure illness which incorporates Chinese herbal medicine (CHM), acupuncture, moxibustion, massage (tui na), and exercise (qi gong). CHM is composed by the principle of “Jun Chen Zuo Shi,” which can achieve the purpose of improving efficacy and reducing toxicity. This combination of compounds is a synergistic effect rather than a simple additional effect. CHM can be also administered in various forms, such as patent medicine, injection, and single-herb extract. In order to clarify the modern theoretical basis and molecular mechanisms of CHM compound and their extractions, great insights have been gained from both in vitro and in vivo studies. This review will focus on the efficacy of CHM in clinical trials and the mechanisms of CHM from the molecular, cellular, genetic, and metabolic levels in the treatment of DKD (Figure 1).

## 2. Traditional Chinese Medicine on DKD

TCM had recorded the literature of DKD, which could be dated back to the Han dynasty (206 BCE to 220 CE) [13]. Over long empirical practice, practitioners have a deeper understanding of the development of DKD and propose the evolution of its pathogenesis. The “Yi-Yang” theory as an ancient Chinese philosophy was applied to clarify human health which is represented as a balance of Yin and Yang in the repeated practice of TCM. Chinese medicine experts speculate that the site of DKD is in the kidney and closely related to the dysfunction of the liver and spleen. The nature of the disease is the intermingled syndrome of deficiency in origin and excess in superficiality. The syndrome of deficiency in origin changes dynamically with the course of disease, which can be divided into three stages, namely, yin deficiency and dry heat in the early stage, qi and yin deficiency in the midterm, and yin damage yang and developing into yang deficiency or both yin and yang deficiency in the later period. The main syndrome of excess in superficiality is categorized into dampness syndrome, blood stasis syndrome, and phlegm syndrome which often coexist and make the disease lingering and difficult to be cured. The treatment principles of DKD are important to tonify the kidney, invigorate qi, nourish yin combined with reduction of phlegm, removal of dampness and blood stasis. On the basis of syndrome differentiation, doctors will employ different treatment principles by individual. Many experienced therapists deem that the pathogenesis of blood stasis runs through the progression of DKD [14]. As a result, activating blood circulation to remove blood stasis should be included in the whole course of preventing and treating of DKD. TCM is not only capable of relieving the local symptoms of disease but also can regulate the integrity of the human body and its interrelationship with the natural environment. There are safety concerns since renal failure cases caused by aristolochic acid (AA) were reported. A systematic review and meta-analysis of randomized placebo controlled trials about CHM for DKD verified that the occurrences of adverse events (digestive disorders, elevated liver enzyme level, infection, anaemia, hypertension, and subarachnoid haemorrhage) were low and CHM appeared to be well-tolerated [15]. A total of 13864 patients of the follow-up study revealed that the use of non-AA prescribed CHMs after the diagnosis of CKD was related to a lower risk of mortality [16].

## 3. The Efficacy of CHM in the Treatment of DKD

Although predominantly regular therapies with tight control of blood glucose and blood pressure levels, strict restriction of protein, and aggressively use of ACEI/ARB are common management strategies for DKD, it is difficult to prevent the onset and/or progress of ESRD. Furthermore, DKD has been the major cause of patient requiring chronic haemodialysis which brings both huge socioeconomic burden and individual burden. Thus, it is a matter of medical urgency to seek valid methods for the treatment of the disease. According to the World Health Organization (WHO), about 80% of the world's inhabitants rely on traditional medicine for primary health care. TCM especially CHM therapy was created by Chinese people in long-term social practice and was proven effective in symptom relief and homeostatic equilibrium. Some randomized clinical trials on the efficacy of CHM treatment of DKD are summarized in Table 1.

A study included 45 early-stage DN patients with the urinary albumin excretion rate (UAER) within 20–200 g/min. Patients were randomly assigned to receive either zishentongluo (ZSTL) (*n* = 25) or benazepril (*n* = 20) for 12 weeks. The primary endpoint (HbA1c) and secondary endpoints (FBG, TC, TG, UAER, Scr, ANP, ET-1, and VEGF) in the ZSTL group were effectively improved compared with the benazepril group. It appears that ZSTL could improve glucose and lipid metabolism and renal function by modulating ET-1, ANP, and VEGF. ZSTL is an effective CHM consisted of *raw astragalus*, *angelica*, *safflower*, *zedoary turmeric*, *Dodder*, *Radix Rehmanniae*, *dogwood*, *Poria*, *Epimedium*, *earthworm*, and *Schisandra* for patients with early-stage DN [17]. Some of the limitations of the study were small sample, short study duration, and no side effects reported during the observation period.

Another study recruited 180 patients with DKD in a six-center, randomized, double-blind, placebo-controlled clinical trial. Based on the conventional treatment with ACEIs or ARBs, 122 participants were randomly assigned to receive Chinese herbal granule Tangshen formula (TSF) and 58 participants to receive placebo for 24 weeks. Primary outcome was the urinary protein level. There was no statistically significant difference in the urinary albumin excretion rate (UAER) for participants with microalbuminuria between the two groups. However, TSF displayed a statistically significant decrease in 24-hour urinary protein (24 h UP) for participants with macroalbuminuria (TSF, 0.21 g compared with placebo 0.36 g). Estimated glomerular filtration rate (eGFR) was improved in both patients with microalbuminuria and macroalbuminuria. TSF (astragalus, burning bush, rehmannia, bitter orange, cornus, rhubarb, and notoginseng) combined ACEI/ARB could not only reduce 24 h UP to a level much lower than that of ACEI/ARB alone but also could improve eGFR in DKD patients with macroalbuminuria. There was no significant difference in the proportions of adverse events between TSF and placebo groups. However, the intervention period was only 24weeks and the long-term hard endpoints, such as doubling of the baseline creatinine level, ESRD, and death, also need to be observed.

Recently, a RCT study recruited 600 type 2 diabetes without diabetic nephropathy (DN) or with early-stage DN which were randomly assigned (1 : 1) to receive Liuwei Dihuang pills (LWDH) and Ginkgo biloba Tablets orally or placebos for 24 months from ten hospitals. The primary outcome was the change of UACR. For patients with UACR ranging 30–299 mg/g at baseline, the reduction of the UACR value between baseline and follow-up was much more obvious in the CHM group compared with that in the placebo group (-25.50 (-42.30, -9.56) vs. -20.61 (-36.79, 4.31), *P* < 0.05). The result suggested that CHM combination to standard clinical intervention appears to be effective for the DN with microalbuminuria [18]. The overall occurrence of adverse events was similar between the two groups. However, there was a relatively high incidence of premature withdrawals, and the follow-up period was too short to evaluate the natural progress of DN.

These established evidence showed that the CHM has a beneficial impact on symptom relief. However, these studies are mostly small size, suboptimal quality. The efficacy on CHM treatment of DKD always applied ACEI/ARB as the positive control group in these clinical studies. Well-designed, large-scale RCT studies need to be carried out with promising agents as control, such as SGLT2 inhibitors.

## 4. The Action Mechanisms of CHM on DKD

TCM has been widely used for treating complex chronic diseases favorable by a great deal of patients owing to abundant experience, expectative efficacy. In order to provide a better clinical service, plentiful experimental researches were carried out to study the mechanisms of CHM. It reveals that CHM exerts a beneficial action on DKD including regulation of metabolic disorders, reduction of oxidative stress, and inhibition activities of AGEs [19, 20]. We will gather some studies related to the regulatory mechanisms of CHM on DKD in Table 2.

### 4.1. Molecular Mechanisms

#### 4.1.1. The Modulation of Metabolic Disorders by CHM

DKD is a metabolic disorder which is characterized by renal damage due to deterioration in insulin secretion or insulin activity. Insulin not only regulates the metabolism of glucose but also directly modulates the biology of podocytes and tubular epithelial cells [21], which are insulin sensitive and express functional insulin receptors [22, 23]. Impaired insulin sensitivity might result in altered renal cell glucose metabolism and also contribute to abnormal vasoreactivity, angiogenesis, and fibrogenesis [24, 25]. In addition, insulin can regulate the flexibility of podocytes and the permeability of GMB, which leads to proteinuria and renal dysfunction. Protein tyrosine phosphatase (PTPase) plays an important negative regulatory role in the intracellular signal transduction pathway, which can make phosphorylated insulin receptor and insulin receptor substrate 1 (IRS1) dephosphorylated, weaken the subsequent insulin signal transduction, and lead to insulin resistance. IRS1 and fibroblast growth factor 21 (FGF21) are two important molecules which can influence insulin signaling [26–28]. *Danhong injection* (*DHI*), a certificated Chinese medical product made from *radix salviae miltiorrhizae* and *flos carthami*, could induce the expression of IRS1, FGF21, and peroxisome proliferator-activated receptor *γ* (PPAR*γ*) in tissues and circulation, which can make contribution to increasing insulin sensitivity and inhibiting the development of DKD [29]. Hu et al. [30] found that *Gosha-jinki-gan* (*GJG*), composed of 10 herb CHM, is not only useful for DKD but also can improve the glucose utilization and insulin resistance in STZ-induced diabetic rats. Lipid deposition can also lead to insulin resistance and restricted the use of glucose capacity in the muscle [31]. *Wen-Pi Tang*, which is composed of five CHM (*Rhei Rhizoma*, *Ginseng Radix*, *Aconiti Tuber*, *Zingiberis Rhizoma*, and *Glycyrrhizae Radix*), showed its protective effects by virtue of the reversal metabolic abnormalities associated with hyperglycemia and hyperlipidemia [32]. The research conducted by Nakagawa et al. [33] indicated that *Keishi-bukuryo-gan* (*Cinnamomi Cortex*, *Hoelen*, *Paeoniae Radix*, *Moutan Cortex*, and *Persicae Semen*) can preserve renal function and ameliorate pathological manifestations through reducing AGE accumulation by virtue of its hypolipidemic effect. *Zhenqing recipe*, containing *Fructus Ligustri Lucidi*, *Eclipta prostrata*, *and Dioscorea opposita*, alleviates DKD by inhibiting the overexpression of sterol regulatory element-binding protein-1c (SREBP-1c) and its target genes including acetyl-CoA carboxylase (ACC) and fatty acid synthase (FAS) which mediated the triglyceride (TG) synthesis pathway in type 2 diabetic rats [34]. Not only the formula preparations but also single herb or its active ingredient has a therapeutical effect on DKD. It is reported that *Corni Fructus* rectified glucose-associated metabolic disorders [35]. *Berberine* (*BBR*, C20H19NO5) is an isoquinoline alkaloid isolated from *Coptidis rhizoma* and *Cortex Phellodendri*, which could serve as a promising approach for treating DKD by improving glucose metabolism disorders, restoring renal functional parameters [36]. *Ginsenoside Rb2* significantly improved glucose tolerance through activation of the NAD-dependent deacetylase sirtuin-1 (SIRT1) and AMP-activated protein kinase (AMPK) signaling pathways [37]. Except for the hypoglycemic or hypolipidemic effects alone, many natural compounds have been shown to balance glucose and lipid homeostasis in diabetes and reduce the incidence of DKD. *Tangningtongluo formula* (*TNTL*), an empirical herb formula of Miao medicine composed of *Plantaginis*, *kewoluoqu*, *Flos Lonicerae*, and *Agrimoniae*, could exert hypoglycemic activity, improve lipid metabolism, ameliorate insulin resistance, and preserve pancreatic *β*-cell damage [38]. Taken together, CHM was beneficial in preventing the progress of DKD by regulating the sensitivity of insulin, the metabolism of glucose and lipid, and the balance of energy homeostasis on multiply target point.

#### 4.1.2. The Modulation of Inflammatory Response

In recent years, more and more evidence confirmed that the abnormalities of immune and inflammatory responses could be the central link of DKD. Genetic factors, metabolism disorders, and hemodynamic changes often act as initiating factors to activate multiple signaling pathways in the kidney tissue. Both the intrinsic cells of the kidney (endothelial cells, mesangial cells, podocytes, and renal tubular epithelial cells) and the foreign cells (macrophages, neutrophils, platelets, lymphocytes, and mast cells) can participate in the inflammatory response of DKD [39]. In addition, there are many proinflammatory cytokines involved, such as chemokines, adhesion molecules, and growth factors [40, 41]. Monocyte chemoattractant protein-1 (MCP-1) is mainly produced by mesangial cells and renal tubular epithelial cells which is a potent chemokine for monocyte/macrophage as well as T cells and is regarded as contributing inflammatory factors for DKD [42]. MCP-1 and tumor necrosis factor-*α* (TNF-*α*) can promote TGF-*β*1 production. Toll-like receptor (TLR) signal transduction, nuclear factor-kappa B (NF-*κ*B), MAPK, and diacylglycerol (DAG)/protein kinase C (PKC) signaling pathways are critical for the regulation of the occurrence of DKD inflammatory response. CHM has an important effect on renoprotection by inhibiting immunity and inflammation response. One study investigated that the pharmacological activity of *Wen-Pi-Tang-Hab-Wu-Ling-San* (*WHW*) extract could largely inhibit the excessive production of inflammatory mediators, NO, TNF-*α*, IL-1*β*, and IL-6, through downregulation of the MAPK and NF-*κ*B pathway [43]. *Shen-Yan-Fang-Shuai* Formula (*SYFSF*) was probably attributable to a renoprotective effect by inhibiting inflammatory response and ECM accumulation mediated by the TNF-*α*/NF-*κ*Bp65 signaling pathway [44]. *SYFSF* was extracted from *Astragali Radix*, *Rheum officinale Baill*, *Radix Angelicae sinensis*, *sargassum*, *Carapax Trionycis*, *Concha Ostreae*, and *Radix rehmanniae preparata*. *Chaihuang-Yishen granule* (*CHYS*), composed of seven herbs (*Radix Bupleuri*, *Radix Astragali*, *Radix Angelicae sinensis*, *Rhizoma Dioscoreae nipponicae*, *Polyporus*, *Folium Pyrrosiae*, and *Hirudo*), inhibited the inflammatory and consequently fibrotic processes by decreasing the protein levels of NF-*κ*B, p65, MCP-1, TNF-*α*, and TGF-*β*1 without affecting the blood glucose level [45]. AGEs also can activate intracellular signal pathways and induce the expression of proinflammatory cytokines in the progression of DKD [46]. *Moutan Cortex* (*MC*), a well-known traditional herbal medicine isolated from *Paeonia suffruticosa Andr.*, could hold a protective effect on inflammation via downregulating the AGE-induced RAGE protein expression and inhibiting the expression of inflammatory cytokines including IL-6, MCP-1, TGF-*β*1, and ICAM-1 [47]. In summary, CHM was found to protect immune-inflammatory pathological injury, which can not only participate in the DKD process as a single mechanism but also serve as the upstream or downstream pathway of other pathogenesis and coordinate with other factors to mediate renal injury [48, 49].

#### 4.1.3. The Modulation of Oxidative Stress

Oxidative stress, one of the pathogenesis of DKD, is also closely related to inflammatory cell recruitment, which results in an increase in inflammation by inducing the production of cytokine, such as IL-1, IL-18, and TNF-*α* [50, 51]. Low level generation of ROS undoubtedly has effects on maintaining HIF*α* stability. However, a large amount of ROS can continuously stimulate HIF*α* and make it slow to respond to hypoxia, resulting in a hypoxic injury of the tubule interstitium. Oxidative stress occurs when the increase of oxygen-free radicals exceeds the antioxidant capacity of our body. On the one hand, the disorder of glucose metabolism can stimulate the body to produce a large amount of ROS, which results in the changes of glomerular vascular permeability and hemodynamics. ROS can directly or indirectly attack the innate cells in the kidney and cause renal fibrosis. In addition, ROS can mediate the occurrence of renal inflammation and accelerate the development of DN. On the other hand, the decreased ability of the body to scavenge free radicals can lead to kidney cell damage. *Paeoniflorin* and *Oxypaeoniflora*, two major compounds in *P. suffruticosa*, could attenuate AGE-induced oxidative damage and inflammation in mesangial cells [52]. One study demonstrated that *Liuwei Dihuang pill* (*LDP*) preserves the function of mesangial cells and prevents the progression of renal fibrosis with multicomponent and multitarget mechanisms involved in antioxidative effects by increasing superoxide dismutase (SOD) and nitric oxide synthase (NOS), decreasing malondialdehyde (MDA) concentrations, and ameliorating the damage caused by lipid peroxidation [53]. SOD is a major macromolecule antioxidant, and its activity represents the capability of tissues to clear oxygen-free radicals. MDA, as a product of lipid peroxidation, reflects the content of oxygen-free radicals in the tissue. The expression level of oxidative carbonyl protein (OCP) could evaluate the degree of ROS-mediated amino acid side chains. *Tripterygium wilfordii Hook. f* (*TWH*) has been used to treat proteinuria for many years in clinical practice, which may have a possible mechanism to reduce proteinuria by downregulation of the expression of OCP in the renal cortex of DKD [54]. *TSF* appeared to be effective in reducing urinary protein and urinary liver fatty acid-binding protein (L-FABP), which was found to be significantly correlated with urinary 8-hydroxy-2′-deoxyguanosine (8-OHdG) levels [55]. Samra et al. found that *Cepharanthine* (*CEP*), *Piperine* (*Pip*), and their combination played a crucial role in inhibiting the inflammatory effect in the DKD animal model through decreasing the thioredoxin-interacting protein (TXNIP) level, acting like a mediator of oxidative stress by directly binding to the antioxidant catalytic site of thioredoxin (TRX), which consequently decrease NLRP3 activation and production of inflammatory cytokines [56]. Free oxygen radicals not only activate macrophage-mediated inflammatory cytokine but also activate transcription factors such as NF-*κ*B [57, 68]. Studies verified that *Danggui-Shaoyao-San* (*DSS*), comprising *Radix Paeoniae Alba*, *Radix Angelicae sinensis*, *Rhizoma Chuanxiong*, *Poria cocos*, *Rhizoma Atractylodis macrocephalae*, and *Rhizoma Alismatis*, had an antidiabetic property and elevated the expression of SOD and glutathione peroxidase (GSH-Px) resulting in downregulation of NF-*κ*B as well as TGF-*β*1 [58]. *Puerarin* exerts the antioxidative effects in podocytes by suppressing the NOX4 expression and upregulating SIRT1, resulting in increased deacetylation of NF-*κ*B [59]. Oxidative stress may also be related to the EMT of tubular epithelial cells, which promote renal interstitial fibrosis in DKD. *Sanziguben Granule* (*SZGB*) can restrain EMT through antioxidative stress effects by stimulating the nuclear factor erythroid-2-related factor 2 (Nrf2) signaling pathway, which is a crucial regulator to counteract oxidative stress and regulate intracellular antioxidants. *SZGB* is a compound prescription made from four kinds of Chinese herbs, namely, *Rosa laevigata Michx*, *Gynostemma pentaphyllum*, *Phyllanthus emblica*, and *Fructus Schizandrae* [60]. CHM can delay the progression of DKD by reducing AGE and the inflammatory response mediated by ROS, regulating the balance of oxidative stress indicators.

#### 4.1.4. The Regulation against Fibrosis

Renal cells produce various growth factors under hyperglycemia condition, especially TGF*β*1, angiotensin II, and platelet-derived growth factor (PDGF), which affect the process of DKD. There are three isoforms, namely, TGF-*β*1, 2, and 3 in the TGF-*β* family. TGF-*β*1 is considered to be the primary factor that drives renal cell hypertrophy and glomerular and tubulointerstitial fibrosis through increasing the expression of ECM proteins, such as collagen and fibronectin [61]. TGF*β*1 can not only bind to the gene promoter by the Smad-dependent pathway to induce the transcription of fibrogenic molecules but also can cross talk with other signaling pathways through non-Smad-dependent pathways, including pp60c-src, epithelial growth factor receptor (EGFR), MAPK, p53, and PI3K/AKT [62] to form a signaling network and jointly enhance the expression of genes related to renal fibrosis [63]. In addition to tissue fibrosis, TGF-*β*1 regulates many biological responses, such as cell proliferation, apoptosis, differentiation, autophagy, and the immune response [64]. A meta-analysis supported that patients with type 2 diabetes mellitus (T2DM) and DKD had increased the level of serum and urine TGF-*β*1 [65]. *Rhodiola rosea* has a protective effect on early nephropathy in diabetic rats by decreasing the TGF-*β*1 expression [66]. *Icariin*, a major constituent of flavonoid, isolated from the plant Herba *epimedii* can evidently relieve renal damage on the early stage of DKD rats by modulating the expression of TGF-*β* protein and collagen IV [67]. A study demonstrated that *astragalus injection* could have an antifibrotic effect by suppressing Smad3, p-Smad3, and the expression of TGF*β*R-I as well as promoting the Smad7 expression [68]. *APF*, the main active constituents extracted from *Xiexin decoction*, can inhibit TGF-*β*1/Smad-mediated fibrosis and reduce NF-*κ*B-dependent inflammation to control renal fibrosis in DKD [69, 70]. Treatment with *Chaihuang-Yishen Granule* (*CHYS*), extracted from *Radix Bupleuri*, *Radix Astragali*, *Rhizoma Dioscoreae nipponicae*, *Polyporus*, *Folium Pyrrosiae*, and *Hirudo*, attenuated diabetic kidney injury through blockade of TGF-*β*1/Smad3-mediated renal fibrosis [71]. TGF-*β*1 not only upregulates genes encoding ECM proteins but also enhances the expression of plasminogen activator inhibitor type 1 (PAI-1) and connective tissue growth factor (CTGF) to further exacerbate ECM production. *Acetylshikonin* from *Zicao* could reduce fibrosis proteins and inhibit TGF-*β*1-induced PAI-1 and collagen III and IV [72]. *Mesona procumbens Hemsl.* could minimize the ultrastructural damages of kidney by reducing the thrombospondin-1 (TSP-1) expression which is a matricellular calcium-binding protein related to plasma proteins (fibrinogen, plasminogen) and matrix proteins (collagens, fibronectin, laminin, and proteoglycans) [73, 74]. Renal fibrosis is a common pathological process from chronic kidney disease to renal failure, which is closely related to the prognosis of DKD. TGF-*β*1 plays a crucial role in the development of renal fibrosis. Studies have shown that CHM can play an antifibrosis role through the intervention of TGF-*β*1 and various fibrogenic cytokine downstream.

### 4.2. Cellular Mechanisms

Renal innate cells include podocytes, mesangial cells, endothelial cells, and tubular epithelial cells. Podocytes cells, endothelial cells, and mesangial cells are mainly involved in the formation of glomerular filtration membrane and the regulation of renal function, while renal tubular epithelial cells mainly take part in the reabsorption and secretion of substances. As the injury target of DKD, innate cells are involved in the occurrence and development of the disease.

#### 4.2.1. The Preservation of Podocytes

Although all cells are chronically exposed to a high glucose process in patients with diabetes, only some show progressive dysfunction leading to renal structural and functional changes. Podocytes are highly specialized, terminally differentiated cells which have an important effect on maintaining the glomerular filtration membrane. Under high blood condition, many factors are known to contribute to podocyte injury, such as AGEs, increased ROS, and activation of the renin-angiotensin-aldosterone (RAAS) system. The pathological characteristics of podocyte injury are mainly manifested as podocyte hypertrophy, abnormal expression of critical proteins in podocyte fissure membrane (nephrin, podocin), podocyte transmembrane proteins (podocalyxin), the effacement and detachment of podocyte foot processes (FPs), and apoptosis of podocytes [75, 76]. *Zhen-wu-tang* (*ZWT*), which is formulated from five herbs including Common Monkshood root, Poria, White Peony root, Atractylodis rhizome, and Zingiberis rhizome, could upregulate the expression of slit diaphragm to possess a renal protective effect [77]. *Qiwei granule* is composed of *Astragalus membranaceus*, *Rehmannia glutinosa*, *Prunella vulgaris*, *Curcuma zedoaria*, *Euonymus alatus*, *Panax pseudoginseng*, and *Rheum officinale*. It could protect the podocyte from developing DKD via increasing the expression of nephrin, CD2AP, and integrin *α*3*β*1 [78]. Wilms tumor 1 (WT1) is a zinc finger-like transcription factor and a podocyte-specific marker which is positive in the podocyte nuclei. The best compatibility of components in *Corni Fructus* (PC) had the protective effect on early nephropathy in type 2 diabetic rats by increasing the expression of WT1 in glomerular podocytes [79]. Podocyte apoptosis is the onset and an early pathological manifestation of DKD. *Catalpol*, a major active ingredient of *Rehmannia*, could ameliorate pathological changes by inhibiting caspase-3 which is a crucial executor or initiating factor of cell apoptosis. *Emodin* protected podocytes from endoplasmic reticulum (ER) stress-triggered apoptosis through the inhibition of the protein kinase RNA-like endoplasmic reticulum kinase (PERK)/eukaryotic initiation factor 2*α* (eIf2*α*) signaling pathway [80]. Podocyte-specific autophagy is a degradation process which contributes to maintaining podocyte function through the clearance of damaged proteins and excess organelles [81]. Elevation of glucose concentration inhibits podocyte autophagy [82]. As a result, autophagy dysregulation is involved in the pathogenesis of podocyte loss, which represents the mechanism of self-renewal in the progression of DKD. Researches provided initial evidence that *hispidulin*, a flavonoid extracted from *Plantago asiatica*, alleviated high glucose-induced podocyte injury by regulating autophagy and Pim1-p21-mTOR signaling axis [83]. It was reported that *mangiferin* could enhance autophagic activity in podocytes by restoring the expression of LC3 II and decreasing the expression of p62. In addition, *mangiferin* might enhance the autophagic process in podocyte via upregulation of AMPK phosphorylation and downregulation of mTOR phosphorylation [84].

#### 4.2.2. The Preservation of Mesangial Cells

Dysfunction of glomerular mesangial cells (GMCs) was also considered to play an important role in the pathogenesis of DKD. GMCs are known to secrete ECM proteins which mainly consist of collagen IV, laminin, and fibronectin. GMC proliferation caused by the accumulation of ECM proteins is a prominent pathological change of DKD. *Dangguibuxue Tang* (*DBT*) is a traditional formulation that is composed of two herbs, *Astragali Radix* and *Angelicae Sinensis Radix*, at the ratio of 5 : 1. One study manifested that it could inhibit high glucose-induced GMC proliferation and synthesis of various ECM proteins, indicating the value for treatment of DKD [85]. Other studies also showed that it could inhibit the proliferation of GMCs by attenuating the *α*-SMA expression and reducing hydroxyproline secretion in cultured GMCs under high glucose conditions [86].

#### 4.2.3. The Preservation of Endothelial Cells

Endothelial cells are activated by high glucose, AGE, and ROS, which recruit and activate mononuclear macrophages and inflammatory signaling pathways, leading to low-grade inflammation. Growing evidence suggests that endothelial dysfunction is closely related to the process of DKD manifesting as endothelial hyperpermeability, disrupted secretion of endothelial-derived vasoactive mediators and phenotype changes [87, 88]. A study indicated that *Salvianolic acid A* ameliorated glomerular endothelial hyperpermeability and alleviated renal structural deterioration through inhibiting the Nox4 and AGE-RAGE-RhoA/ROCK signaling pathway [89].

#### 4.2.4. The Preservation of Epithelial Cells

The transformation of epithelial cells into myofibroblasts (mesenchymal cells) is called renal epithelial-mesenchymal transition (EMT). The features of EMT are characterized by the decreased expression of E-cadherin (E-CA) and the increased expression of *α*-smooth muscle actin (*α*-SMA) in epithelial cells [90]. EMT of tubuloepithelial cells is a widely recognized mechanism that sustains interstitial fibrosis in DKD, which can be regulated by TGF-*β*1 [91]. TGF-*β*1-induced EMT is primarily responsible for ECM accumulation [92, 93]. Researches show that about 30% of fibroblasts are derived from EMT of tubular epithelial cells in the kidney. The preventive activity of herbal mixture of *Radix Puerariae* and *Fructus Crataegi* (*RPFC*) possibly mediated by inhibiting the PI3K/AKT signaling pathway, which indirectly leads to reduction of *α*-SMA and collagen IV [94]. *Tong xinluo (TXL)*, containing 12 medicinal components, could inhibit EMT through decreasing TGF-*β*1 [95]. *Huangkui capsule* alleviates EMT in DKD via inhibiting NLRP3 inflammasome activation and TLR4/NF-*κ*B signaling [96].

Several studies have confirmed that glomerular changes may not only occur in the individual glomerular cells but also have interactions between glomerular cells or through gap junctions. Cross talk among podocytes, endothelial and inflammatory cells mediate mesangial matrix expansion which is associated with the progression of diabetes-associated glomerulosclerosis [97]. The recruitment and maintenance of mesangial cells are dependent on PDGF-B/PDGFR*β* signaling, where PDGF-B is derived from endothelial cells and PDGFR*β* is on the mesangial cells [98]. Conversely, mesangial cells can maintain endothelial cell stability through integrin *α*v*β*8 which can decrease its ligand latent TGF-*β*-mediated signaling in endothelial cells [99]. Podocyte can communicate with endothelial cell through multiple secreted molecules, such as vascular endothelial growth factor (VEGF) [100], Ang-1, Ang-2 [101], and endothelin1 [102]. HGF/c-MET, IGF/IGFBPs, and TNF-*α*/TNFR1 are the major mediators involved in the communication between endothelial cells and podocytes [103, 104]. Identification of the main mediators of cell to cell communication will be valuable for the targeted therapies for DKD. Current studies show that TCM can play a protective role in the innate cells of the kidney by regulating various molecules, signaling pathways, apoptosis, and autophagy processes.

### 4.3. The Regulation of miRNA

MicroRNAs (miR) are highly conserved short-chain noncoding RNAs of approximately 20-25 nucleotides. They play a role in posttranscriptional regulation through binding to the 3′ untranslated region, inhibiting translation or making the target mRNAs degrade to participate in epigenetic interferences of promoting or inhibiting DKD pathogenesis. Recently, many great reports have shown that miRNAs play an essential role in the pathological process of CKD. Several miRNAs have been found to be overexpressed in DKD. In contrast, some miRNAs have been found to be downregulated in DKD, which are inhibitors of DKD-inducing factors, such as TGF-*β*, collagen (COL), NOX, and AKT [105]. For example, high glucose levels promote cell apoptosis by downregulating miR-29a or upregulating miR-29c [106, 107]. More and more studies have shown that miRNAs are widely involved in the development of DKD or could become a new therapeutic target for DKD. miRNAs as a therapeutic target have been focused on modulating several cellular processes in DKD. Lei et al. found that miR-378 was also downregulated in DKD. *Astragaloside* (*AS-IV*) could regulate the miR-378/TNF receptor-associated factor5 (TRAF5) signaling pathway and suppressed subsequently podocyte apoptosis [108]. miR-21 and TGF-*β*1/Smad existed in a complex regulation relationship to enhance renal tubular EMT by inhibiting target Smad7. A research revealed *TXL* ameliorated renal structure and function by regulating miR-21-induced EMT, which might be a key therapeutic target participating in the mechanism of DKD [109, 110]. Han et al. [111] found miRNA-137 is downregulated in diabetic condition which is associated with cell proliferation, invasion, and migration [112]. Importantly, *triptolide* can decrease albuminuria and glomerulosclerosis by upregulating the expression of miR-137 via inhibiting the Notch1 pathway activation [112]. At present, many microRNAs have been found to be closely related to the occurrence and development of DKD, which is expected to be a new biological marker for the diagnosis of DN and a new target for drug therapy. TCM could improve the function and structure of the DKD by a high expression of miRNAs that protect the kidney and the low expression of miRNAs that promote fibrosis.

### 4.4. Metabolism Mechanism

Systems biology is a discipline concept focused on the study of all the interrelationships among genes, proteins, and metabolitesis, which was recognized as a scientific mode and research method in the current study of complex life systems. As one of the related technologies of systems biology, metabolomics has obvious characteristics of reflection thedynamic and integrity. Metabolomics highlights the combination analysis of constituents and pharmacological activities and generates new insights into the researches on the exact target of drug interactions [113], which is highly consistent with TCM holistic view and syndrome differentiation view. The kidney has a very important position in the process of water metabolism in our body. It is often obvious of changes in metabolic substances when the kidney develops a lesion. Therefore, it is of great significance to study the action mechanism of CHM in treating DKD from the perspective of metabolomics. Xiang et al. showed that twenty-one metabolites in serum, sixteen metabolites in urine, and twenty-two in kidney tissues were identified in the DN group compared with the normal group. *Salvia miltiorrhiza* extracts improved the renal injury, and regulation of abnormal metabolism involved pathways of the phospholipid, arachidonic acid, and pyrimidine metabolisms [114]. Dai et al. used the metabolomics method that tentatively identified 27 endogenous metabolites (12 in serum and 15 in urine) involved in sphingolipid metabolism, pentose, glucuronate interconversion, terpenoid backbone biosynthesis, purine metabolism, and retinol metabolism. After the intervention of the total glycosides of RG (TLR) extracted from Rehmannia glutinosa Libosch (RG), twenty-four endogenous metabolites (except for taurochenodesoxycholic acid, chenodeoxycholic acid glycine conjugate, and L-gulonolactone) turned back to a normal level of some extent [115]. Metabolomics, based on the dynamic changes of endogenous metabolites, can be sensitive and real to express the response and alternation of the overall physiological status under various conditions. Studying the effect of CHM on DKD and regulating different metabolites could provide a new method to treat DKD and pave a new way to further explore the pathogenesis of DKD.

## 5. Conclusion

DKD, as a complicated and refractory nephropathy, has the characteristics of multifactorial pathogenesis, which determines that multiple targets should be considered for its treatment. At present, western medicine has been applied to treat DKD mostly focusing on a single site or pathway which makes the effect unsatisfactory. TCM containing different ingredients exhibits distinct advantages with synergistic effects for treatment of DKD compared to chemical agents specific for a single molecular target. It should be noted that we need to avoid drugs containing aristolochic acid when using CHM to treat diseases. With the in-depth researches on the efficacy and action mechanism of TCM in treating DKD, TCM may play a great role in alleviating proteinuria and delaying ESRD. However, there is a need for well-designed, large sample, long-term, randomized, controlled, clinical trials to verify the efficacy and safety of TCM in patients with DKD. It is believed that a new perspective brought by the advanced technology will promote great progress to the research on the CHM treatment of DKD.

## Figures and Tables

**Table 1:** Clinical studies on the efficacy of CHM in the treatment of DKD.

CHM	Composition	*N*	Intervention	Primary outcome	Study period (month)	Outcome
ZSTL	*Raw astragalus*, *angelica*, *safflower*, *zedoary turmeric*, *Dodder*, *Radix Rehmanniae*, *dogwood*, *Poria*, *Epimedium*, *earthworm*, *Schisandra*	45	ZSTL vs. benazepril	Change of HbA1c	3	-4.29% (-5.58, -2.79) vs. -3.26% (-4.06, -1.96) (*P* = 0.0016)
TSF	*Astragalus*, *burning bush*, *rehmannia*, *bitter orange*, *cornus*, *rhubarb*, *notoginseng*	180	Conventional treatment with ACEIs/ARBs added to TSF vs. placebo	Change of urinary protein level	6	-0.21 g (-0.48, 0.06) vs. 0.36 g (-0.04, 0.76) (*P* = 0.024)
LWDH and Ginkgo biloba Tablets	LWDH pills and Ginkgo biloba Tablets	600	CHM treatment vs. placebo	Change of UACR	24	-25.50 (-42.30, -9.56) vs. -20.61 (-36.79, 4.31) (*P* < 0.05)

Abbreviations: ZSTL: zishentongluo; HbA1c: glycated hemoglobin; TSF: Tangshen formula; ACEIs: angiotensin-converting enzyme inhibitors; ARBs: angiotensin receptor blockers; LWDH: Liuwei Dihuang pills; TCM: traditional Chinese medicine; UACR: urinary albumin/creatinine ratio.

**Table 2:** Studies on the regulatory mechanism of CHM in the treatment of DKD.

CHM	Targets	Mechanisms	Ref.	Publication year	Study type
DHI	IRS1, FGF21, and PPAR*γ*	Increasing insulin sensitivity	Liu et al. [29]	2015	Experiment study
Zhenqing recipe	SREBP-1c, ACC, and FAS	Regulating lipid deposition	Wen et al. [34]	2012	Experiment study
SYFSF	TNF-*α*/NF-*κ*Bp65 signaling pathway	Anti-inflammation and ECM accumulation	Lv et al. [44]	2017	Experiment study
MC	RAGE and IL-6, MCP-1, TGF-*β*1, ICAM-1	Anti-inflammation	Zhang et al. [47]	2014	Experiment study
*P. suffruticosa*	CAT and GSH-Px; IL-6 and MCP-1	Antioxidative stress and inflammation	Zhang et al. [52]	2013	Experiment study
TWH	OCP, DMA, SOD	Antioxidative stress	Dong et al. [54]	2017	Experiment study
Puerarin	NOX4, SIRT1, deacetylation of NF-*κ*B	Antioxidative stress	Li et al. [59]	2017	Experiment study
Icariin	Collagen IV, TGF-*β*1; MDA, SOD, Hyp	Antifibrosis and oxidative stress	Qia et al. [67]	2011	Experiment study
Xiexin decoction	NF-*κ*B and TGF-*β*1/Smad signaling pathways	Anti-inflammation and fibrosis	Wu et al. [69]	2015	Experiment study
Zicao	PAI-1, CTGF, collagen III and IV; IL-1*β*, IL-6, MCP-1, ICAM-1	Antifibrosis and inflammation	Li et al. [72]	2018	Experiment study
Qiwei granules	Nephrin, CD2AP, integrin *α*3*β*1; AKT, caspase-3	Protection of podocyte slit diaphragm	Zhou et al. [78]	2015	Experiment study
Emodin	PERK-eIF2*α* signaling pathway	Protecting podocyte apoptosis	Nianxiu et al. [80]	2018	Experiment study
Hispidulin	Pim1-p21-mTOR signaling axis	Alleviating podocyte injury by activating autophagy and inhibiting apoptosis	Wu et al. [83]	2018	Experiment study
Mangiferin	LC3 II, p62, AMPK-mTOR-ULK1 pathway	Protecting podocytes by enhancing autophagy	Wang et al. [84]	2018	Experiment study
DBT	Laminin, collagen IV, and fibronectin	Inhibiting GMC proliferation and ECM proteins	Ke et al. [85]	2012	Experiment study
Salvianolic acid A	AGE-RAGE-Nox4 and AGE-RAGE-RhoA/ROCK signaling pathway	Attenuating oxidative stress, inflammation, and enhancing autophagy of endothelial cell	Hou et al. [89]	2017	Experiment study
TXL	Col IV, FN, TGF-*β*1, E-CA, *α*-SMA	Inhibiting TGF-*β*1-induced EMT	Zhang et al. [95]	2014	Experiment study
Huangkui capsule	NLRP3 inflammasome and TLR4/NF-*κ*B signaling	Regulating renal tubular EMT	Han et al. [96]	2018	Experiment study
Astragaloside	miR-378/TRAF5 signaling pathway	Suppressing the podocyte apoptosis	Lei et al. [108]	2018	Experiment study
TXL	miR-21	Regulating EMT	Wang et al. [110]	2014	Experiment study
Triptolide	miR-137/Notch1 pathway	Preventing ECM accumulation	Han et al. [111]	2017	Experiment study
Salvia miltiorrhiza extracts	Phospholipid, arachidonic acid, and pyrimidine metabolisms	Regulation of abnormal metabolism	Xiang et al. [114]	2019	Experiment study
Rehmannia glutinosa Libosch	Sphingolipid, pentose, glucuronate interconversion, terpenoid backbone biosynthesis, purine and retinol metabolism	Regulation of abnormal metabolism	Dai et al. [115]	2018	Experiment study

Abbreviations: CHM: Chinese herbal medicine; DKD: diabetic kidney disease; DHI: Danhong injection; IRS1: insulin receptor substrate 1; FGF21: fibroblast growth factor 21; PPAR*γ*: peroxisome proliferator-activated receptor *γ*; SREBP-1c: sterol regulatory element-binding protein-1c; ACC: acetyl-CoA carboxylase; FAS: fatty acid synthase; SYFSF: Shen-Yan-Fang-Shuai Formula; TNF-*α*: tumor necrosis factor-alpha; NF-*κ*B: nuclear factor-kappa B; ECM: accumulation of extracellular matrix; MC: Moutan Cortex; RAGE: receptor of advanced glycation end products; IL-6: interleukin-6; MCP-1: monocyte chemoattractant protein-1; TGF-*β*1: transforming growth factor beta1; ICAM-1: intercellular adhesion molecule-1; CAT: catalase; GSH-Px: glutathione peroxidase; TWH: Tripterygium wilfordii Hook. f; OCP: oxidative carbonyl protein; MDA: malondialdehyde; SOD: superoxide dismutase; NOX4: NAPDH oxidase 4; Hyp: hydroxyproline; PAI-1: plasminogen activator inhibitor type 1; CTGF: connective tissue growth factor; PERK: phosphorylated protein kinase RNA-like endoplasmic reticulum kinase; eIF2*α*: eukaryotic initiation factor 2*α*; AMPK: AMP-activated protein kinase; mTOR: mammalian target of rapamycin complex 1; ULK1: Unc-51-like kinase; Col IV: collagen IV; FN: fibronectin; E-CA: E-cadherin; *α*-SMA: *α*-smooth muscle actin; TLR4: Toll-like receptor 4; TRAF: tumor-necrosis factor receptor-associated factor.
